# Metabolomics and Transcriptomics Studies of the Differential Accumulation of Flavonoids in Different Organs in *Emilia sonchifolia*

**DOI:** 10.3390/ijms27156803

**Published:** 2026-07-29

**Authors:** Xuemei Jiang, Rongchang Wei, Yanqing Qin, Jinli Yao, Wenhui Cai, Mingli Huang, Suren Sooranna, Yumei Huang, Liuguan Liang, Jiaxin Wang, Lulu Tan, Chenyan Liang, Liuping Wang, Shan Yang, Dongping Tu

**Affiliations:** 1 College of Pharmacy, Guangxi University of Chinese Medicine, Nanning 530200, China; 18277339890@163.com (X.J.);; 2 Cash Crops Research Institute, Guangxi Academy of Agricultural Sciences, Nanning 530007, China; wrc830612@163.com; 3 College of Chemistry and Chemical Engineering, Central South University, Changsha 410000, China; 4 Department of Metabolism, Digestion and Reproduction, Imperial College London, Chelsea and Westminster Hospital, 369 Fulham Road, London SW10 9NH, UK; 5 Guangxi Botanical Garden of Medicinal Plants, 189 Changgang Road, Nanning 530021, China

**Keywords:** *Emilia sonchifolia*, flavonoids, metabolomics, transcriptomics, UPLC-MS, qRT-PCR

## Abstract

*Emilia sonchifolia* (L.) DC is a medicinal and edible herb of Asteraceae with Lingnan characteristics. Flavonoids are its core pharmacodynamic substances, but the molecular regulation mechanism of differential accumulation of flavonoids in different organs of this species is still unclear. In this study, the molecular basis of tissue-specific synthesis of flavonoids was analyzed by integrating UPLC-MS broad-target metabolome and Illumina high-throughput transcriptome with four tissues of *Emilia sonchifolia*: root, stem, leaf, and flower. The results showed that a total of 73 flavonoid metabolites were identified in the metabolome, including naringenin chalcone, luteolin, quercitrin, and other pharmacologically active substances. Multi-omics joint analysis showed that the floral organ was the core tissue for the synthesis and enrichment of flavonoids, and there were specific characteristic flavonoid subtypes in different tissues. A total of 211 differentially expressed genes related to the flavonoid synthesis pathway were screened by transcriptome analysis, including 16 flavonol synthases, five cinnamic acid 4-hydroxylases, and five chalcone synthases. The WGCNA and gene–metabolite association network showed that the transcription levels of key enzyme genes such as CHS, C4H, F3′H, and F3H were highly positively correlated with the accumulation of downstream flavonols. The qRT-PCR quantitative verification showed that the expression patterns of CHS1, CHI4, F3′H5, F3H, FLS4, and GT in the four tissues were highly consistent with the transcriptome sequencing results, which confirmed that the transcriptome data were reliable. For the first time, this study revealed the molecular regulatory network of tissue-specific accumulation of flavonoids in *Emilia sonchifolia*, and clarified that the flower organ was the optimal medicinal harvesting site of flavonoids. It provided key theoretical support for the breeding of high-efficacy *Emilia sonchifolia* germplasm, the development of flavonoid active ingredients, and the study of secondary metabolic evolution of Compositae plants.

## 1. Introduction

*Emilia sonchifolia* (L.) DC is a traditional Chinese herbal medicine commonly used in southern China. It is composed of the dry whole grass of *E. sonchifolia*. It is named for its red spots in the green clumps and is known by several aliases including Yexiahong, Yangshicao, Yemuercai, Hongbeiye, Honghuacao, and Qishierzhihua. It is used for clearing away heat and detoxification, dispersion of blood stasis and detumescence, and it is a common component of anti-inflammatory and detoxifying Chinese medicines in China [1,2]. The whole plant of *E. sonchifolia* can be used as medicine, and it can be harvested throughout the year. It is a Chinese herbal medicine with extremely high medicinal value, and it is also the main raw material of Huahong tablets and capsules, which are commonly used in gynecological clinics [3].

*E. sonchifolia* is rich in polysaccharides, alkaloids, terpenoids, flavonoids, and other organic substances with potential medicinal value. Among them, flavonoids are the main active components, possessing anti-inflammatory, anti-oxidative, anti-viral, and anti-tumor properties, and the plant is widely used in anti-cancer therapies [4]. Flavonoids can improve hematopoiesis and prevent DNA gene mutation [5]. Previous studies have shown that there are significant differences in the total flavonoid content in the different vegetative organs of *E. sonchifolia*, with the highest amount in the leaves, followed by stems and roots [6]. Shen et al. [7] isolated and identified several flavonoids in the aerial parts of *E. sonchifolia*, including rhamnetin, isorhamnetin, quercetin, luteolin and tricin-7-O-β-D-glucopyranoside. Other studies on the flavonoids of *E. sonchifolia* have focused on content determination [8], the extraction process [9] and activity analysis [10], while the biosynthetic genes in the different parts of the plants have not been elucidated.

The combined analysis of integrated metabolome and transcriptome has become the mainstream paradigm for analyzing the regulation of secondary metabolism in medicinal plants, which effectively breaks through the technical limitations of single transcriptome or metabolome that can only unilaterally describe molecular or metabolic characteristics and cannot analyze causal regulatory relationships [11]. As an important secondary metabolite of plants, the biosynthetic pathway of flavonoids has been relatively clear, starting from the phenylpropanoid pathway, and then catalyzed by chalcone synthase (CHS) and chalcone isomerase (CHI) to form the core skeleton of flavonoids [12]. In the downstream branch pathway, flavonol synthase (FLS), isoflavone synthase (IFS), and anthocyanin synthesis-related enzymes (ANS/UGT) further regulate structural diversity [13]. Recent studies have revealed the regulation of the MYB-bHLH-WD40 transcription complex and epigenetic mechanisms on the pathway [14]. Metabolic engineering was used to achieve efficient synthesis of flavonoids in crops and microorganisms [15]. Some researchers have identified key enzymes such as CmCHS and CmFNS and MYB and bHLH to regulate the differential accumulation of flavonoids by WGCNA-associated Chrysanthemum morifolium petal development stage genes and flavonoids and chlorogenic acid metabolites, and MYB and bHLH regulate the differential accumulation of flavonoids [16]. Studies have combined transcriptome and extensive targeted metabolomics to analyze the differential accumulation of flavonoids in multiple tissues of *Panax japonicus*, identify nine key synthetic genes and regulate WRKY transcription factors, and elucidate the regulatory mechanism of flavonoid spatial synthesis [17]. However, the current research system for *E. sonchifolia* still stays at the phenotypic and macro-functional levels, and there are still obvious knowledge shortcomings, which is difficult to support the accurate development of its active ingredients and molecular breeding optimization.

In this study, we analyzed the differences in the composition and content of flavonoids in different tissues of *E. sonchifolia*, focusing on the key enzyme of flavonoid biosynthesis by using transcriptomics. The internal reference genes stably expressed in *E. sonchifolia* were screened by quantitative real-time-PCR (qRT-PCR) [18], and we determined the tissue-specific expression of the differentially expressed genes (DEGs) of these enzymes. This study provides a scientific basis for further studies on the synthesis and metabolism of flavonoids in *E. sonchifolia*, in order to improve the production of its bioactive metabolites. It can serve as a guide to increase the efficiency of harvesting *E. sonchifolia* and improve the production of this widely used resource.

## 2. Results

### 2.1. Analysis of the Flavonoids of E. sonchifolia

**Figure 1:**
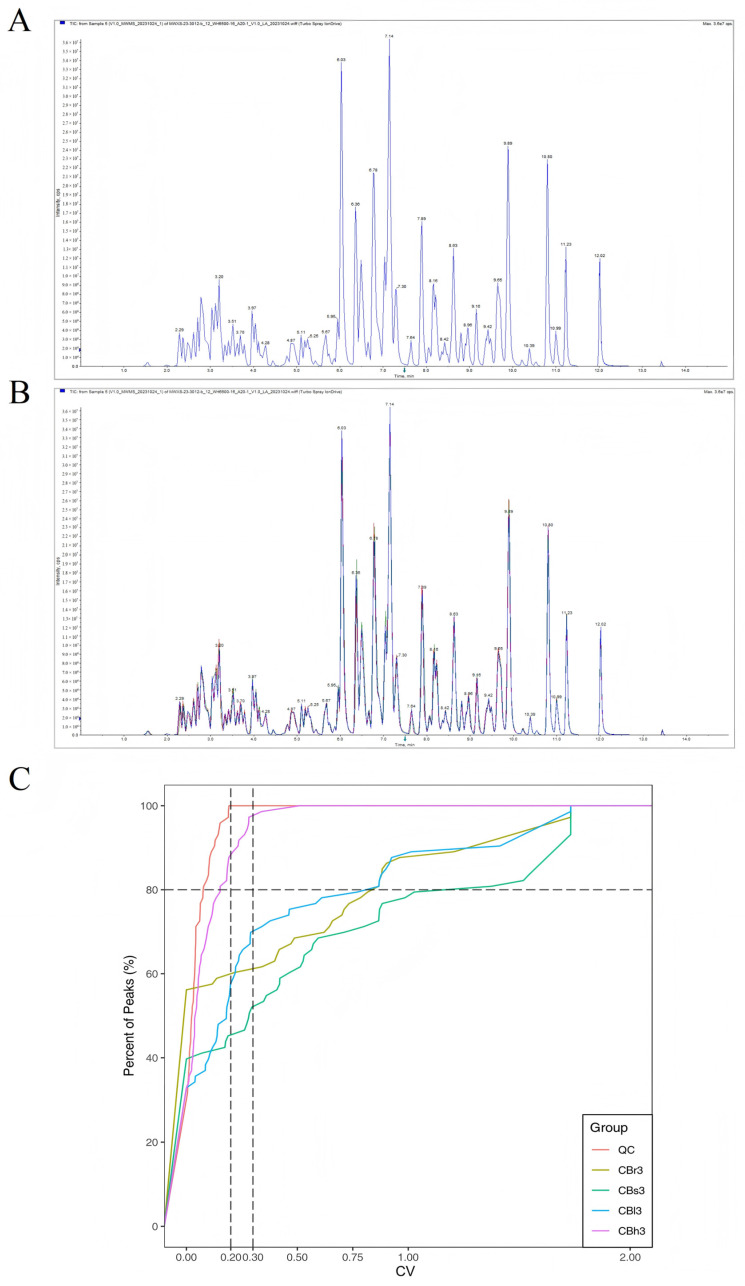
Qualitative and quantitative analysis of red metabolome. (**A**) Total ion current diagram. (**B**) TIC overlap diagram. (**C**) CV distribution in each group of samples. “QC” is the quality control sample, “CBr” is the sample of roots, “CBs” is the sample of stems, “CBl” is the sample of leaves, “CBh” is the sample of flowers.

From the total ion current (TIC) spectra of various quality control samples detected by mass spectrometry (Figure 1A,B), it can be seen from the analysis of overlapping display technology that the curve is highly overlapped, the signal intensity is stable, and the system error is very small. The empirical cumulative distribution function (ECDF) is used to analyze the frequency of CV of substances less than the reference value. The proportion of substances with CV less than 0.2 in QC samples is higher than 80%, indicating that the experimental data are very stable (Figure 1C).

A total of 73 flavonoid metabolites were identified (Supplementary Table S2) by using the metabolomics data. In total, 32, 44, 49 and 18 were detected in roots, stems, leaves and flowers, respectively. These included 18 flavonoids, 21 flavonols, six dihydroflavones, seven dihydroflavonols, eight chalcones, seven isoflavones, three flavanols, one phenolic acid, one xanthone, and one other flavonoid (Supplementary Figure S1). There were several types of flavonoids and flavonols, accounting for 24.66 and 28.77% of the total, respectively, and these were the main metabolites of *E. sonchifolia*.

**Figure 2:**
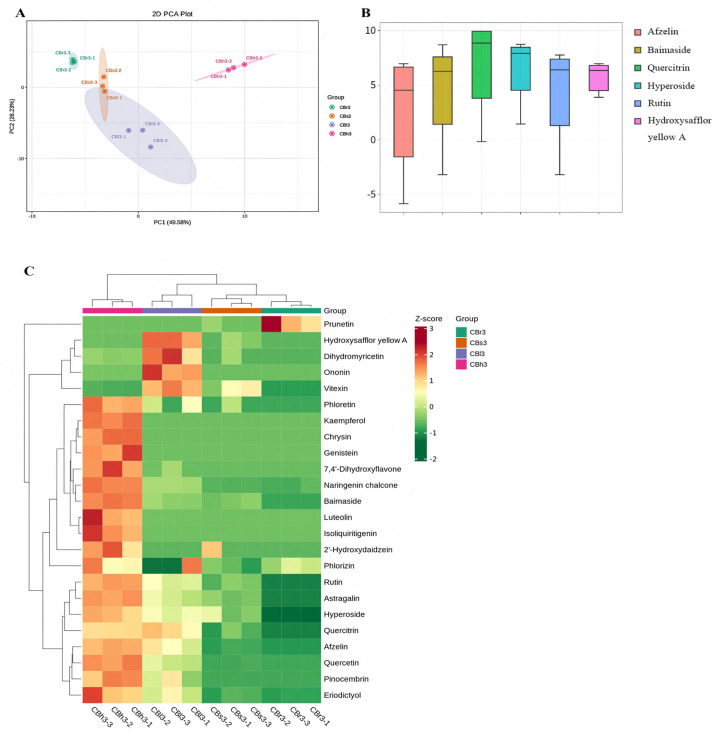
(**A**): A principal component analysis chart of the different tissues of E. sonchifolia. Note: PC1 is the first principal component, PC2 is the second principal component, and percentage is the interpretation rate of the principal component to the dataset; each point in the Figure represents a sample, and the different colors represent different samples, with the same color representing the biological repetition in the same group. (**B**): The main flavonoid metabolites in different parts of *E. sonchifolia*. (**C**): A heatmap of the flavonoid-related differential metabolites in different parts of *E. sonchifolia*.

Principal component analysis of the samples showed that the projection areas of stem and leaf metabolites partially overlapped, indicating that there was little difference between them. The first principal component (PAC1) accounted for 49.58% of the total variance, separating the *E. sonchifolia* flowers (CBh3) from the leaves (CBl3), stems (CBs3), and roots (CBr3). The second principal component (PAC2) accounted for 26.23% of the total variance of *E. sonchifolia*, which separated CBr3 from other tissues (CBh3, CBl3 and CBs3). The principal component analysis showed good repeatability between the samples and that there were differences in metabolites between the samples in different parts (Figure 2A).

Quantitative analysis of flavonoid metabolites in the samples of *E. sonchifolia* (Supplementary Table S2) showed that quercitrin was the highest flavonoid differential metabolite detected in the different parts of *E. sonchifolia*, with an average value of 974.55 nmol/g in flowers, followed by hyperoside, baimaside, rutin, hydroxysafflor yellow A, and afzelin. Among these compounds, hydroxysafflor yellow A had the highest content in the stems, with an average value of 143.48 nmol/g, and the other flavonoid metabolites were highest in the flowers. Quercitrin accounted for the largest proportion (55%) in the total content of flavonoid metabolites in leaves, followed by flowers (44%), stems (38%), and roots (6%). Compounds such as astragaloside, syringaldehyde, and hyperoside were the main components of *E. sonchifolia* roots, accounting for 88% of the total. Quercitrin, hyperoside, cannabinoid, rutin, hydroxysafflor yellow A, vitexin, and afzelin were the main components in the stems, accounting for 98% of the total. Quercitrin, hyperoside, hydroxysafflor yellow A, rutin, cannabinoid, and afzelin were the main components in leaves, accounting for 97% of the total. Quercitrin, hyperoside, sesamin, rutin, kaempferol-3-rhamnoside, luteolin, kaempferol-3-O-β-D-rutinoside, hydroxysafflor yellow A, apigenin, narcissin, and other compounds accounted for 98% of the total in the flowers (Figure 2B). The selected differential metabolites were mapped into a cluster heatmap (Figure 2C). It was found that most of the metabolites were higher in the flowers, including 19 metabolites such as naringenin chalcone, luteolin, quercetin, kaempferol, and eriodictyol. It may be closely related to reproductive adaptability, such as pollen fertility maintenance, flower color presentation, and pollinator attraction. Hydroxysafflor yellow A, dihydromyricetin, formononetin, vitexin, and four metabolites were higher in the stems, and only one cherry yellow metabolite was higher in the roots.

**Figure 3:**
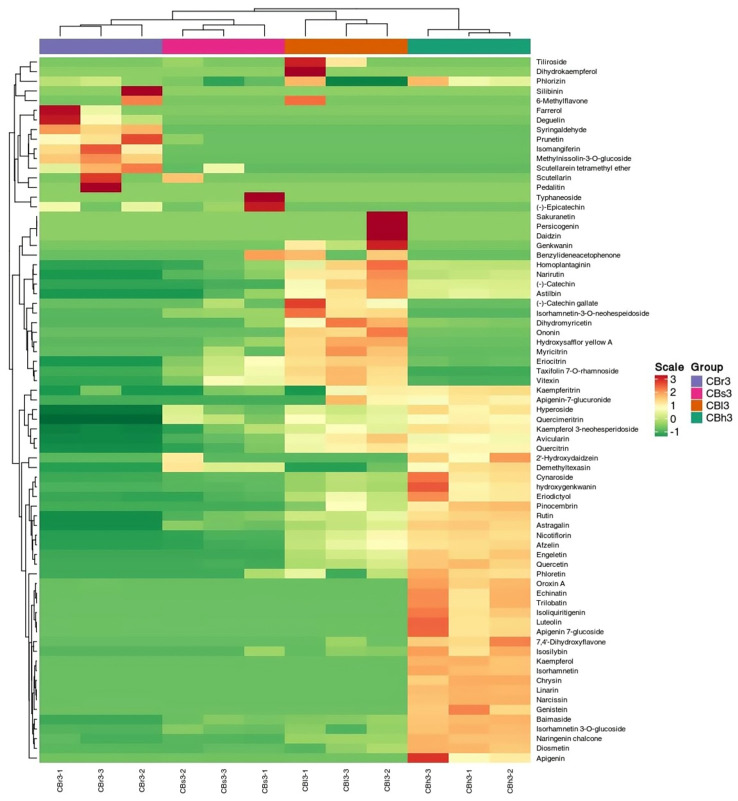
Cluster analysis of the flavonoid metabolites. Note: Cluster analysis was performed on the metabolites as well as the samples. The horizontal row refers to the sample name, and the vertical column refers to the metabolite. The gradation of the different colors refers to the different values obtained after standardization (with red and green representing high and low, respectively; the left clustering line in the graph is the metabolite clustering line, and the clustering line above the graph refers to the sample clustering line).

**Figure 4:**
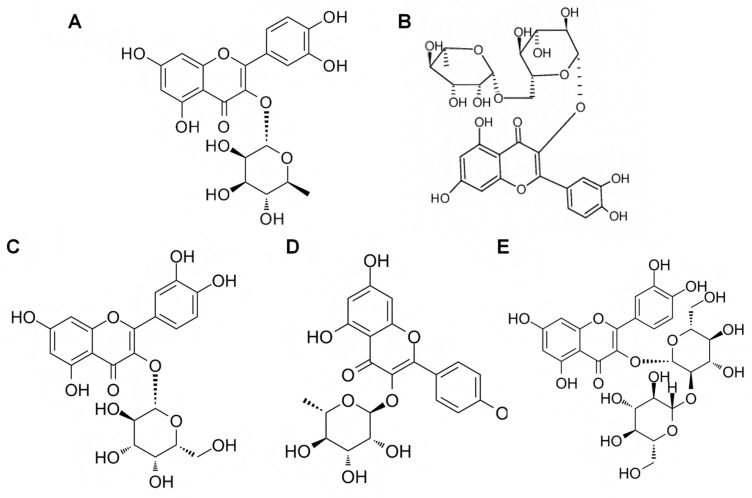
The chemical structural formula of the main metabolite of *Emilia sonchifolia*: (**A**) Quercitrin; (**B**) Rutin; (**C**) Hyperoside; (**D**) Afzelin; and (**E**) Quercetin 3-O-sophoroside.

The contents of 73 flavonoid metabolites detected were standardized by Z-score and presented in the cluster heat map (Figure 3). The chemical structure of the main metabolites is shown in Figure 4.The results showed that there were significant differences in metabolic profiles among roots, stems, leaves, and flowers. Among them, the accumulation of 68.5% flavonoid metabolites in floral organs, such as apigenin 7-glucoside, quercetin derivative (Hyperoside), and kaempferol 3-neohesperidoside, was significantly higher than that in other parts, especially in flavonoid glycosides. In contrast, the roots are specifically enriched in isoflavones such as Echinatin and Genistein, while the stems show a unique distribution pattern of dihydroflavonoids such as Dihydromyricetin. Notably, core flavonoid skeleton components such as catechin ((−)-Catechin) and apigenin were detected in all parts, but their contents in flowers were 2.3–4.1 times higher than those in roots, stems, and leaves, respectively. It is suggested that the floral organ may be the core site of flavonoid biosynthesis, and its high expression characteristics may be related to the strong activation of the phenylpropanoid metabolic pathway during reproductive development.

### 2.2. The Differential Accumulation of Flavonoids

A total of 57 flavonoid DAMs were screened by analysis. In the CBh3 vs. CBr3 comparison group, the number of flavonoid differential metabolites was the highest, with 44, of which 42 metabolites increased and two decreased. In the CBh3 vs. CBs3 comparison group, there were 38, of which 37 increased and one decreased; in the CBh3 vs. CBl3 comparison group, there were 32, of which 23 increased and nine decreased; the number of differential metabolites of flavonoids in the CBr3 vs. CBs3 comparison group was the least, which was 24, of which five metabolites increased and 19 decreased. In the CBr3 vs. CBl3 comparison group, there were 34, of which five increased and 29 decreased; in the CBs3 vs. CBl3 comparison group, there were 26, one increased and 25 decreased (Supplementary Figure S2).

In order to analyze the specific distribution of metabolites in different tissues of *Emilia sonchifolia*, the top 20 metabolites in the Log 2 FC of six groups were visualized (Supplementary Figure S3). The results showed that flavonols were the most significant metabolite groups in all comparison groups. Rutin, quercetin, kaempferol, luteolin, and their glycoside derivatives were at the forefront of up-regulation in flowers, and the accumulation of flavonoids in flowers was significantly higher than that in roots, stems, and leaves. When comparing roots with stems and stems with leaves, flavonoids were gradually up-regulated in stems and leaves, showing a gradient accumulation characteristic from roots to stems and leaves to flowers.

### 2.3. Functional Annotation and Enrichment Analysis of Differential Flavonoids-Metabolites

**Figure 5:**
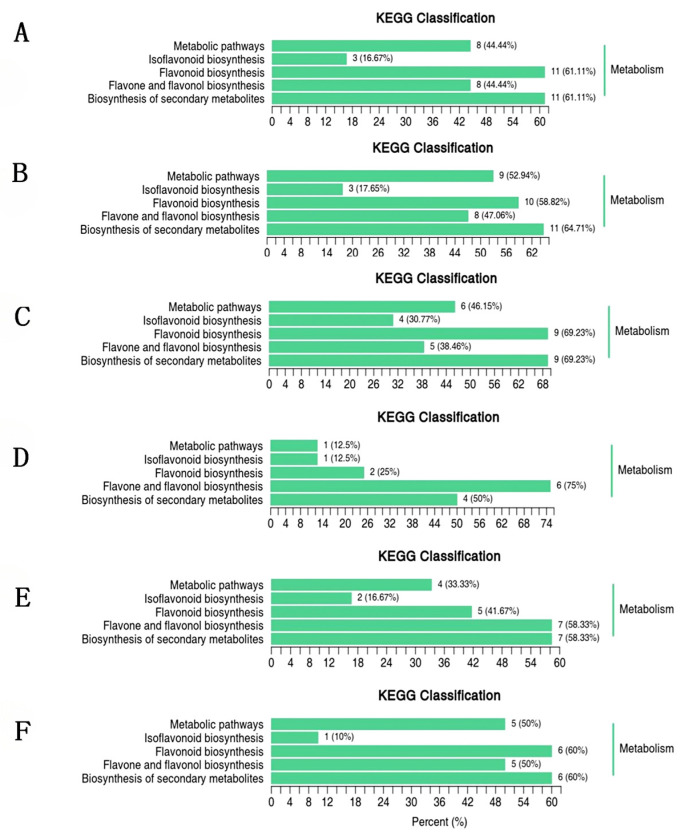
KEGG enrichment of differential metabolites in *Emilia sonchifolia*: (**A**) KEGG histogram of differential metabolites in CBr and CBh comparison groups; (**B**) KEGG histogram of differential metabolites in CBl and CBr comparison groups; (**C**) KEGG histogram of differential metabolites in CBs and CBr comparison groups; (**D**) KEGG histogram of differential metabolites in CBh and CBl comparison groups; (**E**) KEGG histogram of differential metabolites in CBh and CBs comparison groups; and (**F**) KEGG histogram of differential metabolites in CBl and CBs comparison groups.

All the differential metabolites in different parts of *E. sonchifolia* were annotated to the KEGG pathway for enrichment analysis, and they were shown to enrich in five pathways. These included the metabolic pathway (ko01100), biosynthesis of secondary metabolites (ko01110), flavonoid biosynthesis (ko00941), flavone and flavonol biosynthesis (ko00944), and isoflavone biosynthesis (ko00943). The biosynthesis of flavonoids was highly enriched in the comparison groups of CBh3 vs. CBs3, CBh3 vs. CBr3, CBh3 vs. CBl3, and CBs3 vs. CBl3, which accounted for more than 58% of the pathways. The biosynthesis of flavonoids and flavonols was highly enriched in the comparison group of CBr3 vs. CBs3 and CBr3 vs. CBl3, accounting for 75% and 58.33%, respectively, of the total (Figure 5). In the KEGG pathway annotation (ko00941, ko00944, ko00943) and the literature support, we first set the significance threshold fold change ≥ 2 and *p* < 0.05, screening out the initial candidate metabolites, and then, through the KEGG mapping, identified three core pathways: 13 kinds of flavonoid biosynthesis, six kinds of flavonoid/flavonol synthesis, and five kinds of isoflavone synthesis. Finally, combined with literature verification, the metabolites with species characteristics or clear biological activity were preferentially retained, and 24 key differential metabolites of flavonoids were identified from different parts of *E. sonchifolia* by multi-stage screening.

### 2.4. Construction of a Library and Gene Annotation

The Illumina platform was used to perform transcriptome sequencing on different parts of *E. sonchifolia*. In total, 80.21 Gb of clean data was obtained after quality evaluation. The clean data from each sample reached 6 Gb, the percentage of Q30 bases was 93% or above, and the GC content was higher than 41.36% (Supplementary Table S3). The results showed that the filtered sequencing data were of high quality and could be used for subsequent gene expression analysis. A total of 119,886 unigenes were obtained after assembly of the clean data, of which there were 30,081 unigene fragments that were greater than or equal to 2000. All unigenes were compared and annotated by the Kyoto Encyclopedia of Genes and Genomes (KEGG), the non-redundant protein database (NR), the protein sequence database (Swiss-prot), the protein eukaryotic homology database (KOG), the gene ontology joint database (GO), the protein family (PFAM) database, along with other databases. Among them, the NR and TrEMBL databases annotated the most relevant information, with 84,534 and 84,466 annotations, respectively, accounting for more than 70% of the total clean data (Table 1). These annotation results provided gene function and classification information, and the sample sequencing assembly results were qualified, which provided the basic data for subsequent analysis.

### 2.5. Differentially Expressed Gene Analysis

**Figure 6:**
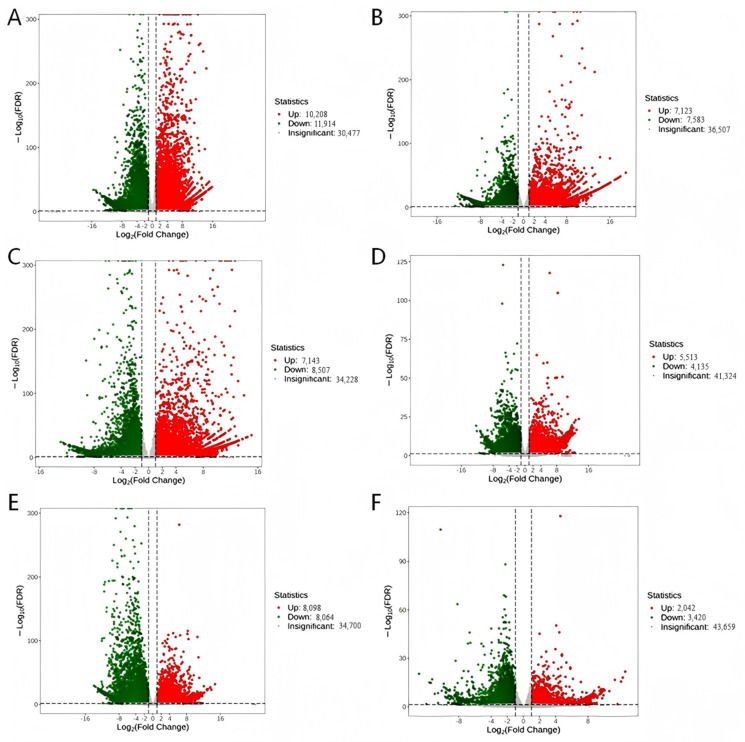
Volcano maps of the differentially expressed genes in different sites of *E. sonchifolia*. Note: (**A**) CBh3 vs. CBr3; (**B**) CBh3 vs. CBs3; (**C**) CBh3 vs. CBl3; (**D**) CBr3 vs. CBs3; (**E**) CBr3 vs. CBl3; (**F**) CBs3 vs. CBl3.

A total of 32,797 DEGs were obtained from analysis of *E. sonchifolia*, and the significant differences between the groups are shown in the volcano maps (Figure 6). The number of DEGs in the comparison group of CBh3 vs. CBr3 was the largest, with 22,122, of which 10,208 and 11,914 were significantly up- and down-regulated, respectively (Figure 6A). There were 14,706 genes in the CBh3 vs. CBs3 group, of which 7123 were significantly up-regulated, and 7583 were significantly down-regulated (Figure 6B). There were 15,650 genes in the CBh3 vs. CBl3 group, of which 7143 and 8507 were significantly up- and down-regulated, respectively (Figure 6C). There were 9648 genes in the CBr3 vs. CBs3 group, of which 5513 and 4135 were significantly up- and down-regulated, respectively (Figure 6D). In the CBr3 vs. CBl3 and CBs3 vs. CBl3 groups, there were 16,153 and 5462 genes, of which 8089 and 2042 were significantly up-regulated, and 8064 and 3420 were significantly down-regulated, respectively (Figure 6E,F). A large number of differentially expressed genes between the two comparison groups of each tissue fully proved that there was a dramatic transcriptome differentiation in the roots, stems, leaves, and flowers of *E. sonchifolia*, and the difference in gene expression between flowers and roots was the most prominent. The genes related to the flavonoid synthesis pathway were differentially transcribed in different organs and finally drove the tissue-specific enrichment of flavonoid metabolites.

**Figure 7:**
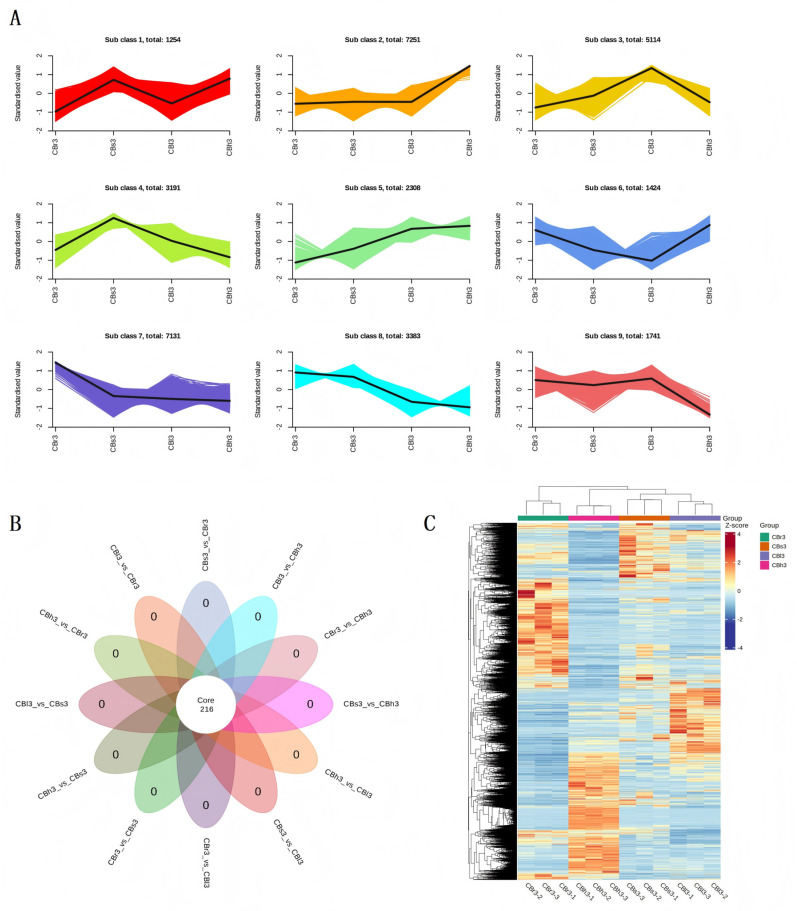
Cluster analysis of differential gene expression. (**A**) K-means clustering diagram. The abscissa represents the sample, and the ordinate represents the standardized expression. (**B**) Differential gene Wayne diagram. (**C**) All comparison groups and set differential gene clustering heat map.

The results showed that these genes were divided into nine expression patterns by K-means clustering of standardized expression levels, showing tissue-specific high expression characteristics of roots, stems, leaves, and flowers, respectively. Wayne analysis showed that there were no unique differential genes among all the comparison combinations, and only 216 core genes showed differences in all tissue comparisons. The clustering heat map further verified that the samples were clearly clustered according to the tissue type, and the gene expression pattern was highly consistent with the K-means results, which intuitively showed the overall distribution and specific expression characteristics of differential genes in different organs (Figure 7).

### 2.6. Screening of DEGs Related to Flavonoid Synthesis Pathway

**Figure 8:**
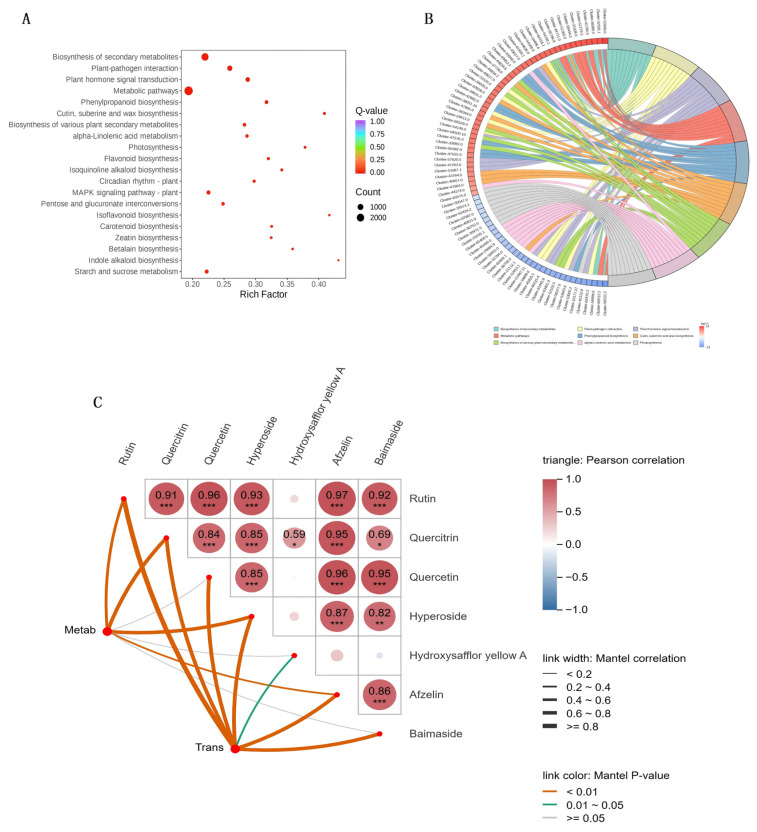
(**A**) KEGG enrichment scatter plot of the top 20 items with the most significant enrichment (**B**) KEGG enrichment chord diagram. The right side of the figure is the nine most significant enrichment pathways, and the left side is the 10 genes with the largest difference in the multiple |logFC|values in each pathway. The middle line represents the correspondence between the pathway and the gene, and the lower right heat map legend represents the logFC value of the gene. Red represents up-regulated genes, blue represents down-regulated genes, and the shade of color represents the size of the logFC values. The darker the color, the greater the difference in multiples. (**C**) Analysis of the association network between the *E. sonchifolia* transcriptome (Trans) and metabolome (Metab). * means *p* < 0.05, ** means *p* < 0.01, *** means *p* < 0.001.The line represents Mantel correlation, and the line width represents the correlation coefficient. Orange represents *p* < 0.01, green represents *p* in 0.01~0.05, gray represents *p* ≥ 0.05.

The degree of KEGG enrichment was measured by Rich factor, Qvalue and the number of differentially expressed genes enriched in this pathway. The 20 pathway entries with the most significant enrichment were selected for display. The larger the Rich factor, the greater the degree of enrichment. The smaller the Qvalue, the more significant the enrichment.(Figure 8A). KEGG enrichment analysis was performed on the differentially expressed genes, and the string graph was drawn. The results showed that the nine most significant pathways were enriched, including phenylpropanoid biosynthesis, flavonoid biosynthesis, plant hormone signal transduction, etc. There is a clear correspondence between the core genes with the largest difference in the multiple |logFC| values in each pathway. These pathways cover secondary metabolic synthesis, hormone regulation, stress response, basic energy metabolism, etc. There is an *E. sonchifolia* core biological process (Figure 8B).

According to the analysis of the correlation network between the transcriptome (Trans) and the metabolome (Metab), it was found that most metabolites such as rutin, quercitrin, and quercetin were significantly positively correlated with the transcriptome (Pearson correlation coefficient 0.84–0.97, *p* < 0.01), and the Mantel correlation width was ≥0.8, indicating that the accumulation of these flavonoid metabolites was highly synergistic with the expression of related genes. Only hydroxysafflor yellow A was not significantly associated with the transcriptome (*p* ≥ 0.05), suggesting that its synthesis may be regulated by other pathways or at non-transcriptional levels, and this transcription–metabolism association result was also indicated. The expression changes in key enzyme genes (such as CHS, F3H, FLS, etc.) in flavonoid synthesis in the transcriptome can directly drive the accumulation of flavonoid metabolites. Subsequently, the ‘gene–metabolite’ regulatory network of *E. sonchifolia* flavonoids can be further clarified through KEGG pathway enrichment (Figure 8C).

In total, 496 differential genes related to the biological metabolism of flavonoids were screened from the transcriptome data of the sample by KEGG differential gene enrichment analysis. When combined with the flavonoid metabolic pathways enriched by differential metabolites, the differential genes annotated by the flavonoid biosynthetic pathway (ko00941, ko00943 and ko00944) of the samples were further screened, and 211 DEGs were obtained. A total of 28 enzyme genes were included, such as the key enzymes in flavonoid metabolic pathways, such as *C4H*, *CHI*, *CHS*, *F3′H*, and *FLS* (Supplementary Table S4). Moreover, 162, 16, and 48 unigenes were mapped on the flavonoid, flavonol, and isoflavone synthetic metabolic pathways, respectively. Among them, ko00941 and ko00943 co-annotated flavonoid synthase II, ko00941 and ko00944 co-annotated flavonoid-3′-monooxygenase, and ko00941 and ko00940 co-annotated trans-cinnamic acid ester-4-monooxygenase. Multi-pathway synergistic division of labor regulates the differential synthesis of various flavonoid components and elucidates the transcriptional regulation basis of tissue-specific accumulation of *E. sonchifolia* flavonoid metabolites at the molecular level.

### 2.7. Transcription Factor Analysis

**Figure 9:**
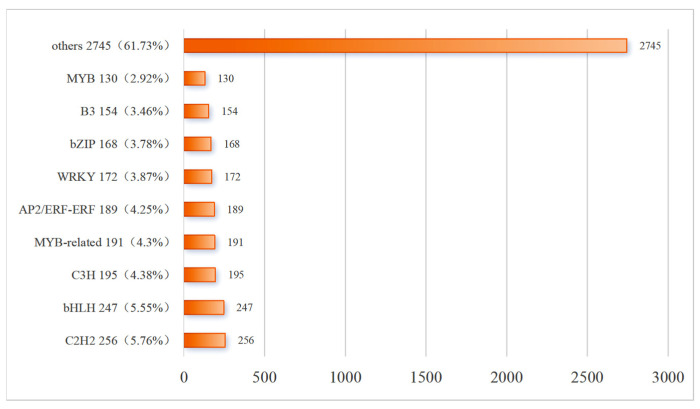
Transcription factor annotation classification statistics of *E. sonchifolia*.

In the analysis of the *E. sonchifolia* transcription factor family, the annotated transcription factors were mainly ‘others’ (2745, accounting for 61.73%). The top nine transcription factor families included C2H2 (256, 5.76%), bHLH (247, 5.55%), C3H (195, 4.38%), MYB-related (191, 4.30%), AP2/ERF-ERF (189, 4.25%), WRKY (172, 3.87%), bZIP (168, 3.78%), B3 (154, 3.46%), and MYB (130, 2.92%). Among them, MYB, bHLH, WRKY, and other transcription factor families closely related to flavonoid biosynthesis have high abundance, suggesting that these family members may play an important role in the transcriptional regulation network of flavonoid biosynthesis by directly or indirectly regulating the expression of key enzyme genes (Figure 9).

### 2.8. Comprehensive Analysis of Metabolomics and Transcriptomics

**Figure 10:**
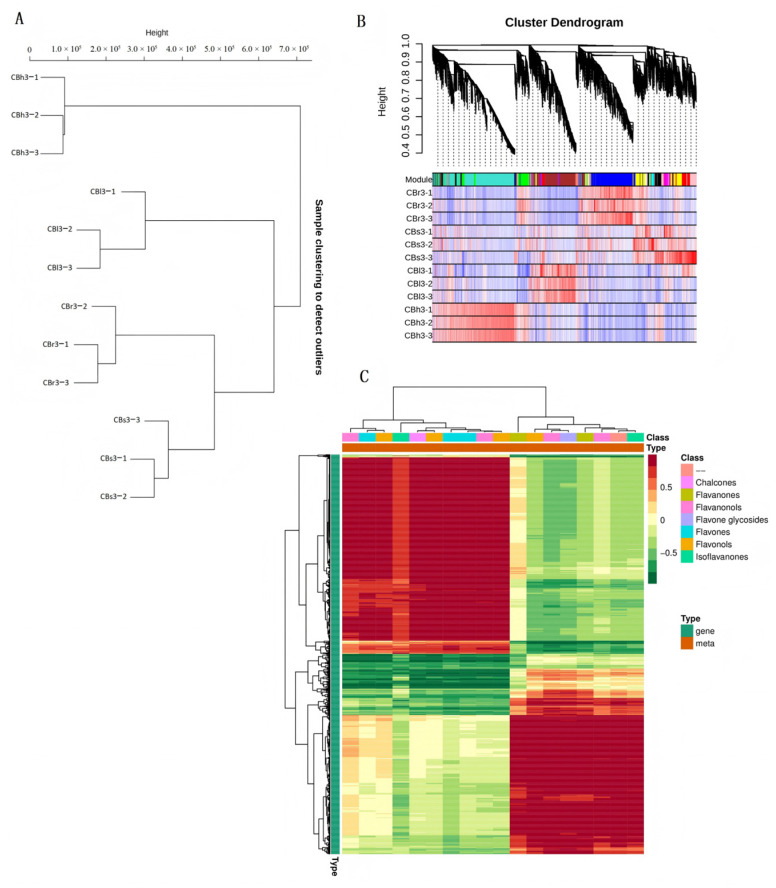
(**A**) Sample clustering tree. (**B**) The upper part is the gene clustering tree. The middle part corresponds to each module, and the lower part is the gene expression clustering heat map. (**C**) Cluster heat map of correlation between differential genes and differential metabolites. Red represents a positive correlation between genes and metabolites, and green represents a negative correlation between genes and metabolites.

Through the comprehensive analysis of different tissue samples, firstly, based on the transcriptome data, all samples were hierarchically clustered (Figure 10A). The results showed that the biological duplicate samples of the same tissue were clustered into one group, and there was no outlier sample, indicating that the experimental repeatability was good and the sample grouping was reasonable. On this basis, the expressed genes were divided into multiple independent modules (Figure 10B) by WGCNA, and the gene expression patterns in each module were highly similar. Further gene–metabolite association heat map (Figure 10C) analysis showed that there was a significant co-expression correlation between differentially expressed genes and different types of flavonoid metabolites, including chalcone, flavanone, flavonoid glycosides, flavonoids, flavonols, and isoflavones, showing obvious module differentiation characteristics. Some gene clusters were strongly positively correlated with metabolites, such as chalcone and flavonoid glycosides, and negatively correlated with flavonoids and flavonol metabolites. The other gene clusters showed the opposite correlation pattern, which was positively correlated with flavonoids and flavonol metabolites, and negatively correlated with chalcones, suggesting that these genes may participate in the regulation of different branches of the flavonoid synthesis pathway through different regulatory pathways.

**Figure 11:**
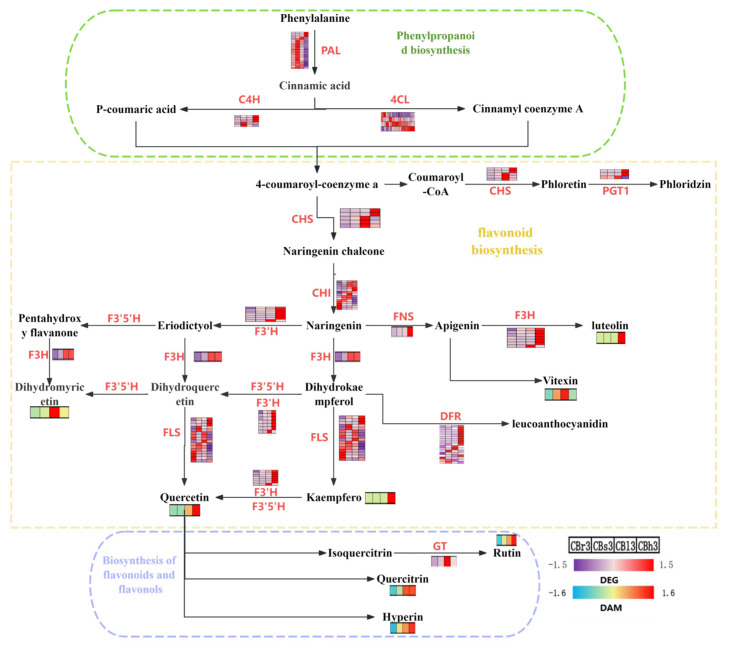
A heatmap of the flavonoid biosynthesis pathway of *E. sonchifolia*. Note: *PAL*: phenylalanine ammonia lyase; *C4H*: cinnamic acid-4-hydroxylase; *4CL*: 4-coumaric acid CoA ligase; *CHS*: chalcone synthase; *CHI*: chalcone isomerase; F3′H: flavonoid 3′-hydroxylase; *F3′5′H*: flavonoid 3′,5′-hydroxylase; *F3H*: flavonoid 3-hydroxylase; *FLS*: flavonol synthase; *FNS*: flavonoid synthase; *PGT1*: phloridzin synthase; *GT*: flavonol-3-O-glucoside L-rhamnosyltransferase.

The differential metabolites and genes co-enriched by metabolomics and transcriptomics KEGG pathways were analyzed for the regulatory network of flavonoid accumulation (Figure 11). This revealed the regulatory relationship between the expression of key enzyme genes and the levels of the related differential metabolites. In total, 14 differential metabolites were enriched in the flavonoid metabolism-related pathways in different parts of *E. sonchifolia*, which were naringenin chalcone, phloretin, genistein, 7,4′-dihydroxyflavone, eriodictyol, luteolin, dihydromyricetin, vitexin, quercetin, kaempferol, afzelin, rutin, quercitrin, and hyperoside. A total of 115 differential genes were selected. These included 12 key enzyme genes: phenylalanine ammonia lyase (*PAL*, 23), cinnamic acid-4-hydroxylase (*C4H*, 5), 4-coumarate CoA ligase 4 (*4CL*, 22), chalcone synthase (*CHS*, 5), chalcone isomerase (*CHI*, 14), flavonoid 3′-hydroxylase (*F3′H*, 7), flavonoid 3-hydroxylation (*F3H*, 1), flavonol synthase (*FLS*, 16), flavonoid synthase (*FNS*, 1), dihydroflavonol 4-reductase (*DFR*, 17), phloridzin synthase (*PGT1*,3) and flavonol-3-O-glucoside rhamnosyltransferase (*GT*, 1). A heatmap of the flavonoid synthesis metabolic pathway in *E. sonchifolia* showed that the differential metabolites and genes of flavonoid biosynthesis showed different accumulation patterns in roots, stems, leaves, and flowers. In addition, some differential genes showed the same expression trend as the downstream metabolites. Among them, the metabolites naringenin chalcone and phloretin were significantly accumulated in the flowers, followed by the leaves, and less accumulated in the roots. Metabolome–transcriptome joint association analysis showed that the organ-specific expression of core enzyme genes such as PAL, CHS, and FLS directly drives the tissue-specific distribution of flavonoid metabolites such as naringenin chalcone.

The regulatory trends of differential genes, *C4H* and *CHS*, were similar. The differential genes, *CHI* and *FLS*, were significantly expressed in different parts of *E. sonchifolia*, and the expression levels in leaves were relatively high. The differential gene *F3′H* showed the highest accumulation in the flowers, followed by the leaves, and the expression trends of the upstream metabolite, kaempferol, and downstream metabolite, eriodictyol, were similar. The expression of the differential gene, *F3H*, was higher in the flowers and stems, and the downstream metabolite product, dihydromyricetin, was higher in stems, which was consistent with the regulation trend of *F3H*. The expression levels of *GT* in the leaves were higher than those in the flowers, but the accumulation of its downstream product, rutin, in the flowers was higher than that in the stems, and the expression levels were inconsistent. The reason may be due to the long-distance transport of rutin across tissues through the vascular system, and the directional transport of rutin generated by leaves as a synthetic source to the storage of floral organs, resulting in inconsistent gene expression and metabolite accumulation levels. It may also be the tissue-specific regulation of GT protein translation efficiency, stability, and catalytic activity by post-transcriptional regulation. The high abundance of GT mRNA in leaves makes it difficult to be converted into active enzymes with catalytic function, resulting in limited in situ rutin synthesis. The amounts of eriodictyol, luteolin, kaempferol, quercetin, quercitrin, rutin, and hyperoside were all high in the flowers. The high expression levels of F3′H, F3H, and CHS in flowers were positively correlated with the significant accumulation of metabolites such as eriodictyol and quercetin (*p* < 0.05), while the expression peaks of CHI, FLS, and GT genes in leaves or stems were partially temporally or spatially mismatched with the cross-tissue distribution of downstream products such as rutin (r = 0.65–0.78). It is worth noting that these genes are widely expressed in roots, stems, leaves, and flowers (TPM > 15), suggesting that these genes may be partially involved in the tissue-specific regulation of flavonoid metabolites, but their specific mechanism of action needs to be further verified by protein activity and metabolic flux analysis.

### 2.9. qRT-PCR Expression Analysis of Key Enzyme Genes

**Figure 12:**
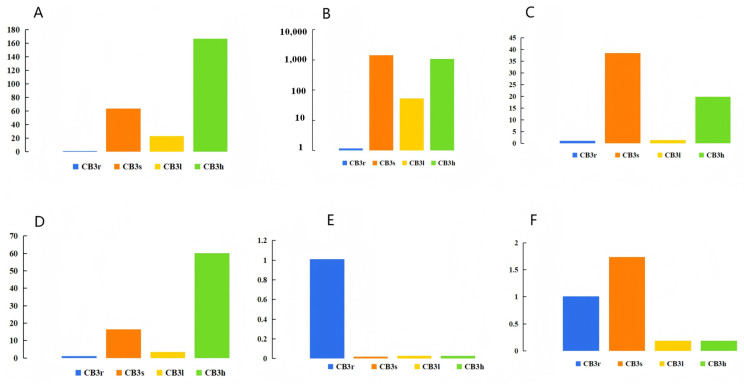
Relative expression of the key enzyme candidate genes. Note: (**A**): *CHI4*; (**B**): *CHS1*; (**C**): *F3H*; (**D**): *F3′H5*; (**E**): *GT*; (**F**): *FLS4*.

Transcriptome differential expression analysis was performed using |log2FC| > 1 and FDR < 0.05 as a screening threshold to screen tissue-specific high-expression key enzymes CHS, CHI, F3′H, F3H, FLS, and GT. Then, combined with the expression levels of each gene sequence in each key enzyme in different tissues, six key enzyme gene sequences with the highest expression levels were finally determined: CHS1, CHI4, F3′H5, F3H, FLS4, and GT. Since F3H and GT only contain a single key enzyme gene sequence, no further screening was performed, and qRT-PCR experiments were performed on the internal reference gene UPL and the six key enzyme genes involved in the biosynthesis of flavonoids in different parts of *E. sonchifolia* (Figure 12). The results showed that the expression trend of key enzyme candidate genes in roots, stems, leaves, and flowers was consistent with the sequencing results, and the relative expression was lowest in the roots. The relative expression levels of *CHS1*, *CHI4*, *F3′H5*, and *F3H* in the stems and flowers were higher, and those of *CHI4* and *F3′H5* in the flowers were the highest, which verified the reliability of transcriptome sequencing data.

## 3. Discussion

There are about 50 species in the genus Emilia, but there are few studies on the flavonoid spectrum of other representative species in the genus. In this study, the accumulation patterns and molecular regulatory networks of flavonoids in roots, stems, leaves, and flowers were systematically compared for the first time by integrating metabolomics and transcriptomics analysis. A total of 73 flavonoid metabolites were identified by metabolomics, of which 24 key differential metabolites (such as quercitrin, luteolin, naringenin chalcone, etc.) were significantly enriched in the flavonoid biosynthesis pathway (ko00941), flavonoid and flavonol biosynthesis pathway (ko00944), and isoflavone biosynthesis pathway (ko00943). *E. sonchifolia* has been used as a whole herb with rich pharmacological activities. It has antibacterial, anti-inflammatory, analgesic, anti-oxidation, immune enhancement, hypoglycemic, anti-tumor, and other effects [19,20,21]. Among them, flavonoids are its main active ingredients [22]. As early as 2012, Shen Shoumao et al. [23] studied the chemical constituents of the aerial parts of *E. sonchifolia* and isolated quercetin, luteolin, and other compounds. Quercetin is a flavonoid monomer compound widely found in plants, with anti-tumor, anti-inflammatory, anti-oxidation, and hypoglycemic effects [24]. In this study, the quantitative analysis of metabolomics showed that most of the flavonoid metabolites were higher in flowers, including quercitrin, luteolin, kaempferol, naringenin chalcone, etc. At present, in addition to quercetin, rutin, afzelin, etc., 48 metabolites such as daidzin and phloretin are reported for the first time to separate potential new components in *E. sonchifolia*, which can be used as candidate targets for subsequent LC-MS targeted screening and column chromatography separation. The principal component analysis showed that the content of flavonoids in flower organs was significantly higher than that in other tissues. In particular, the average content of quercitrin in flowers was 974.55 nmol/g, accounting for more than 44% of the total flavonoids. These preliminary results indicated that the highest distribution of flavonoids was in flowers. It is speculated that flowers are the main medicinal parts of *E. sonchifolia*, which provides a direction for guiding the harvest of medicinal materials and giving full play to the efficacy of *E. sonchifolia*.

It has been reported that the vegetative organs and floral organs of *Tagetes patula* generally contain basic flavonoids such as quercetin [25], and enrichment of Bidens alba flavonoids in aerial tissues [26]. The results of this study showed that the flavonoid components of *Emilia sonchifolia* had significant tissue-specific distribution characteristics, and the flavonoid accumulation level in floral organs was significantly higher than that in vegetative organs such as stems and leaves. The results of this study showed that the flavonoids of *Emilia sonchifolia* had significant tissue-specific distribution characteristics, and the accumulation of flavonoids in flower organs was significantly higher than that in stems and leaves. This unique metabolic distribution pattern shows potential chemotaxonomic characteristics, which further confirms the uniqueness of *Emilia sonchifolia* in the allocation strategy of flavonoid metabolic organs, and provides an important basis for the study of chemical classification, phylogenetic evolution, and secondary metabolic diversity of *Emilia sonchifolia*. Previous studies have shown that rutin, quercetin, and kaempferol are the main flavonol components of parsley plants [19], and the results of this study are consistent with them. The floral organ is a sensitive reproductive tissue, and long-term natural selection promotes *Emilia sonchifolia* to preferentially allocate metabolic resources to the flower and enrich flavonols to protect the reproductive structure. The stems and leaves rely on morphology and basic metabolism to resist stress, and the accumulation level of flavonoids is lower, forming the distribution characteristics of flavonoid-specific enrichment in flower organs. This differential metabolic allocation is an evolutionary strategy for the adaptation of *Emilia sonchifolia* to the environment and also provides theoretical support for the screening of medicinal parts and the development of flavonoid resources. The accumulation of flavonoids in floral organs is an adaptive metabolic feature formed by the long-term evolution of *Emilia sonchifolia*. A high content of flavonols can effectively absorb ultraviolet light and reduce the oxidative damage of germ cell DNA, which may be the key to ensuring the pollination and fruiting of *Emilia sonchifolia* [27]. The content of flavonols in flowers may also be able to regulate the coloration of petals and assist in attracting pollinators, thereby improving the success rate of plant reproduction. Studies have shown that flavonoids have significant inhibitory activity against fungi and bacteria, thereby reducing the risk of infection of floral diseases during flowering [28]. Therefore, it is possible to promote the synthesis of flavonoids in petals by regulating light, water, and fertilizer conditions during the cultivation of *Emilia sonchifolia*, which may not only reduce flowering diseases and reduce the cost of plant protection drugs but also improve the pollination and fruit setting rate, improve plant breeding yield and seedling cultivation efficiency.

The distribution of flavonoids in different tissues of plants is significantly different. The important reason for this tissue difference is that the enzyme content and activity related to the metabolism of flavonoids have tissue differences [29]. Metabonomics and transcriptomics were integrated to analyze the pathway relationship between differential metabolites and differential genes in the flavonoid biosynthetic pathway of *P. multiflorum*. Six key enzyme candidate genes (*CHS*1, *CHI*4, *F3′H*5, *F3H*, *FLS*4, *GT*) involved in the flavonoid metabolic pathway were preliminarily screened. The expression trends of different parts of *E. sonchifolia* were basically consistent with the expression trends of metabolites. Through the analysis of transcription factor annotation, the core transcription factor families MYB, bHLH, and WRKY involved in the regulation of flavonoid biosynthesis in *Emilia sonchifolia* have high abundance. The MYB family (including MYB-related) [30] is the core transcription regulator of plant flavonoid synthesis, and its family members can directly activate or inhibit the expression of downstream genes by identifying the cis-acting elements on the promoters of key enzyme genes for flavonoid synthesis, such as PAL, CHS, CHI, and FLS. At the same time, MYB protein often interacts with bHLH family members to form an MBW (MYB-bHLH-WD40) ternary complex, which synergistically enhances the transcriptional regulation ability of the flavonoid biosynthesis pathway [31]. The high abundance distribution of the bHLH family in this study further confirms the potential importance of this complex in the regulation of flavonoid biosynthesis.

CHS and CHI are key enzymes in the flavonoid synthesis pathway and play an important regulatory role. Their gene expression levels are related to metabolite production [32]. F3′H5 is one of the key enzymes regulating the hydroxylation mode of the flavonoid B ring, which mainly plays a regulatory role in flower color modification and resistance to stress [33]. In addition, the expression of the F3H gene is inhibited or overexpressed, which will directly lead to the down-regulation or up-regulation of flavonoid metabolism and synthesis, and can participate in the regulation of flavonoid resistance to abiotic stress [34]. Studies have shown that dihydroflavonol promoted by F3H plays an important role in the response of Dendrobium officinale plants to stress [35,36]. In this study, CHI, as the rate-limiting enzyme of flavonoid skeleton synthesis, was the most active in leaves, and CHS was the most active in flowers, which was consistent with the accumulation trend of downstream metabolites. F3′H and F3H significantly affected the conversion of dihydroflavonol to flavonol by regulating the hydroxylation mode of the B ring, and their high expression in flowers was synchronized with the accumulation of kaempferol and eriodictyol. FLS is also one of the key enzymes in the synthesis of flavonols. It is highly expressed in leaves, but its catalytic product kaempferol is more abundant in flowers, suggesting that there may be a cross-tissue transport or enzyme activity regulation mechanism [37,38,39]. The expression level of GT in leaves is higher than that in flowers, but the accumulation of its downstream product rutin is higher in flowers than in stems [40]. The anti-inflammatory activity of rutin is better than that of quercetin, and the presence of glucosyl and rhamnosyl has anti-inflammatory activity.

qRT-PCR showed that the expression trends of the key enzyme candidate genes in the roots, stems, leaves, and flowers were basically consistent with the sequencing results, which suggested that the sequencing results obtained were reliable. However, there is a slight inconsistency between transcriptome data and qRT-PCR results in gene expression patterns such as CHS and CHI, which may be due to the multiple complexities of technical methods and biological backgrounds. On the one hand, the high-throughput characteristics of RNA-Seq may be limited by batch effects, gene isomer annotation bias, or low-abundance transcript detection sensitivity. Although the targeting of qRT-PCR can accurately quantify specific transcripts, it is more sensitive to primer specificity, internal reference gene stability, and sample processing timeliness. On the other hand, post-transcriptional regulatory mechanisms such as mRNA stability, translation efficiency, or tissue-specific subcellular localization differences may lead to a decoupling of transcript abundance and protein functional activity. In order to reconcile this contradiction, it is suggested to expand biological repetition to exclude individual heterogeneity, integrate metabolomics data to explore the dynamic correlation between gene expression and metabolic flux, and re-evaluate the potential interference of experimental conditions such as sampling time points on the two technologies. Future research needs to analyze the spatial and temporal specificity of ‘transcription–translation–function’ cascade regulation under the framework of multi-omics, so as to more comprehensively explain the biological logic of gene expression pattern and phenotype association.

## 4. Materials and Methods

### 4.1. Sample Collection

**Figure 13:**
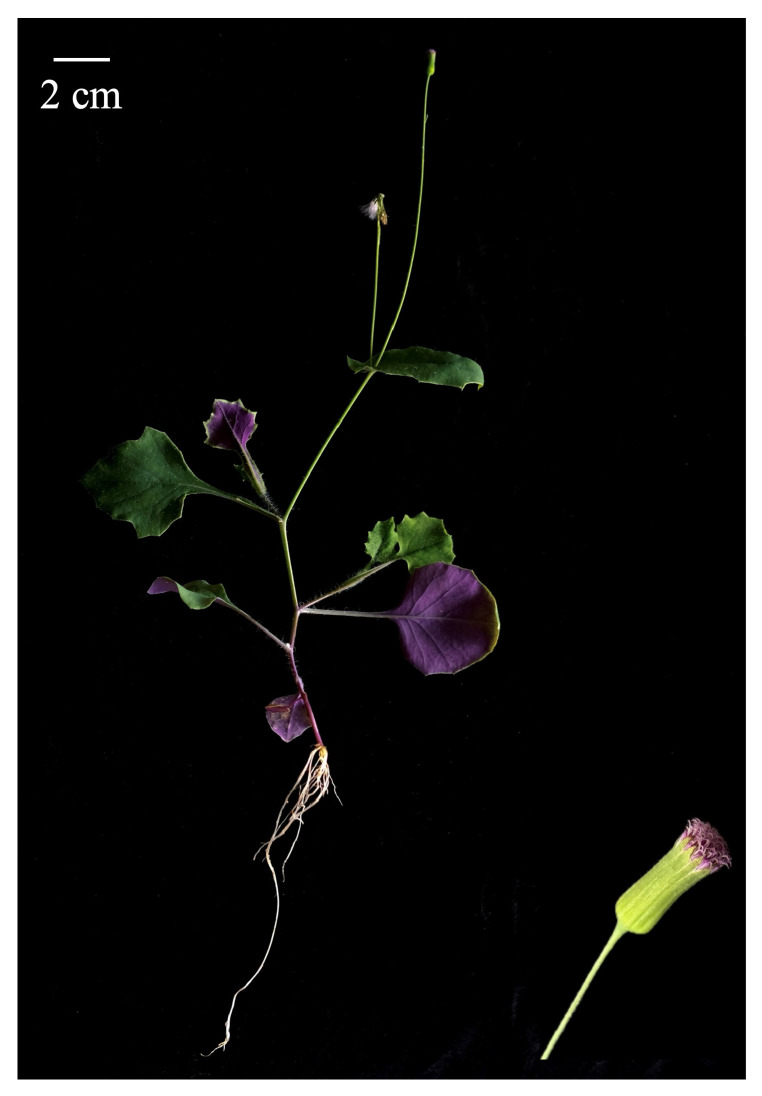
*Emilia sonchifolia* original plant.

The samples of this study were collected in the summer of August 2023 in the Qingxiu District, Nanning City, Guangxi Zhuang Autonomous Region, at a longitude of about 108.5045° E and a latitude of about 22.7988° N. The soil was loose and humid, the temperature was about 32 °C, and the altitude was about 100 m. The roots, stems, leaves, and flowers of three positive flowering plants with the same growth size were selected as repeated samples. Each tissue had three samples, and all samples were used for RNA sequencing. The numbers allocated to the samples were as follows: CBr3-1 (root), CBr3-2 (root), CBr3-3 (root), CBs3-1 (stem), CBs3-2 (stem), CBs3-3 (stem), CBl3-1 (leaf), CBl3-2 (leaf), CBl3-3 (leaf), CBh3-1 (flower), CBh3-2 (flower), and CBh3-3 (flower). After harvesting, the samples were preserved at a low temperature, and they were returned to the laboratory for cleaning with deionized water. Samples were taken from different parts of roots, stems, leaves, and flowers. The samples were placed in liquid nitrogen for quick freezing for 5–10 min, and then they were stored at −80 °C for later use. They were sent to Wuhan Metware Biotechnology Inc. for flavonoid quantitative metabolomics measurements and transcriptome sequencing. The *Emilia sonchifolia* (L.) DC specimen was collected in the Herbarium of Guangxi University of Traditional Chinese Medicine. The collection number was 20235090002, and the collector was Yao Jinli. All samples were identified as the whole plant of *Emilia sonchifolia* (L.) DC by Professor Dongping Tu of Guangxi University of Chinese Medicine (Figure 13).

### 4.2. Metabolomic Sequencing of E. sonchifolia

The samples of root, stem, leaf, and flower of *E. sonchifolia* were dried by vacuum freezing, and then they were ground for 1.5 min to powder at 30 Hz using a grinding instrument. The internal standard of Wuhan Maiwei Metabolic Biotechnology Co., Ltd. (Wuhan, China) was adopted; that is, the company uses the internal standard to correct according to the samples provided by us. The selected internal standard is generally the isotope internal standard or structural analog of the substance to be detected (Supplementary Table S5). In total, 20 mg of powder was weighed, and 10 μL of internal standard at a working solution of 4 mmol/L and 500 μL of 70% methanol solution were added, and the mixture was sonicated for 30 min. The samples were centrifuged at 4 °C and 12,000 rpm for 5 min, and the supernatant was filtered through a 0.22 μm filter membrane. The samples were stored in the injection bottle and analyzed by ultra-high performance liquid chromatography-mass spectrometry (UPLC-MS). The UPLC analysis conditions were: Waters ACQUITY UPLC HSS T3 C18 column (1.8 μm, 100 mm × 2.1 mm); the mobile phase A was added with 0.05% formic acid, and the mobile phase B was added with 0.05% formic acid. The elution gradient was 0 min A/B 90: 10 (*v*/*v*), 1 min A/B 80: 20 (*v*/*v*), 9 min 30: 70 (*v*/*v*), 12.5 min A/B 5: 95 (*v*/*v*), 13.5 min 5: 95 (*v*/*v*), 13.6 min 90: 10 (*v*/*v*), 15 min 90: 10 (*v*/*v*). The flow rate was 0.35 mL/min, and the column temperature was maintained at 40 °C. The injection volume was 2 μL.

The mass spectrometry analysis conditions used were electrospray ionization (ES) at a temperature of 550 °C, mass spectrometry at a voltage of 5500 V (positive ion mode), and mass spectrometry at a voltage of 4500 V (negative ion mode), with curtain gas (CUR) at 35 psi. In the triple quadrupole (QQQ), each ion pair was scanned and detected based on the optimized de-clustering potential (DP) and collision energy (CE). Subsequently, the Metware database (MWDB) constructed by Wuhan Metware Biotechnology Co., Ltd. (Wuhan, China) standardized the original readings by the normalization method and corrected the sequencing depth of each sample, and the statistical model was used to calculate the hypothesis test probability (*p*-value). Finally, multiple hypothesis test correction was performed to obtain the FDR value (false discovery rate), and, finally, the flavonoid components in *E. sonchifolia* root, stem, leaf, and flower were qualitatively and quantitatively analyzed. After using Analyst 1.6.3 software to process the mass spectrometry data, the chromatographic peaks detected in different samples were corrected by MultiQuant 3.0.3 software. The detected peak area ratio obtained was substituted into the standard curve linear equation for calculation, which ultimately resulted in the content data of the metabolites in the samples.

### 4.3. Transcriptomic Sequencing of E. sonchifolia

The total RNA of roots, stems, leaves, and flowers was extracted using a polysaccharide polyphenol plant total RNA extraction kit. The integrity of RNA and the presence of DNA contamination were assessed by agarose gel electrophoresis. The RNA concentration was measured using a Qubit 4.0/MD microplate reader (Thermo Fisher Scientific, Waltham, MA, USA), and for the integrity of RNA, a Qsep400 biological analyzer (BiOptic, New Taipei City, Taiwan) was used. Following assessment, a library was constructed, and this was qualified; then, an Illumina platform was used for sequencing. The raw reads were obtained, and the fastp software (0.23.2) was used to control the quality of the data, and some of the reads were removed. After removal of the N content that exceeded 10% of the read base number, those with low quality (Q ≤ 20) that exceeded 50% of the read base number were the high-quality clean reads were obtained. These were then assembled using Trinity software (v2.13.2). DIAMOND BLASTX software (v2.0.9) was used to compare the unigene sequences obtained with the KEGG NR, Swiss-Prot, GO COG/KOG, and TrEMBL databases.

After predicting the amino acid sequences of the unigenes, HMMER software (3.3.2) was used to compare the data obtained with the PFAM database to obtain their annotation information. The expression levels of transcripts were calculated by using fragments per kilobase of transcript per million fragments (FPKM). A differential analysis was performed using DESeq2 software (1.22.2), and the false discovery rate (FDR) was obtained. The screening criteria for DEGs were set as |log2Fold Change| ≥ 1 and FDR < 0.05. After that, the selected DEGs were compared with the KEGG database for functional annotation and enrichment, and to analyze and predict their functions and the metabolic pathways they were involved with.

### 4.4. Combined Analysis of Metabolomics and Transcriptomics

Based on the screening results of differential genes and differential metabolites in flavonoid metabolic pathways, through multi-dimensional bioinformatics analysis, including FPKM standardization of gene expression profiles and LC-MS/MS mass spectrometry peak area normalization calculation of metabolites, the transcriptome sequencing data and metabolomics characteristic peak clustering analysis results were co-located in the KEGG database (ko00940, ko00941, ko00943, ko00944). Cytoscape platform (3.10.4) was used to construct the metabolic network map of flavonoids. Combined with weighted gene co-expression network analysis (WGCNA) and metabolite ion flow fingerprint (EIC) technology, the regulatory relationship between gene expression and metabolite accumulation was revealed. The spatial distribution characteristics of tissue-specific metabolomics and transcriptome sequencing data were further integrated, and the differential accumulation of flavonoids in different tissues and organs (roots, stems, leaves, and flowers) and its molecular regulation mechanism were elucidated by system biology methods. It provides an important theoretical basis for further understanding the biosynthetic pathway of flavonoids and their tissue-specific accumulation.

### 4.5. qRT-PCR Verification of the Key Enzyme Genes

According to the literature research and the data of the *E. sonchifolia* transcriptome, the DESeq2 algorithm (|log2FC| ≥ 1.5, *p*adj < 0.01) was used to screen the core regulatory gene clusters of the flavonoid biosynthesis pathway. Combined with the metabolomics data, MapMan 3.6.0 was used to carry out hierarchical clustering analysis of metabolic pathways, and a pathway map based on Z-score standardized metabolite content and FPKM gene expression was constructed. Finally, four internal reference genes, Tubulin, Ubiquitin, GAPDH, and UPL, were selected as candidate genes, and six key enzyme genes involved in the synthesis of *E. sonchifolia* flavonoids were selected. Primer Premier 5.0 software was used to design primers (Supplementary Table S1) according to their CDS sequences. The gene sequences were synthesized by Nanning Dennis Biotechnology Co., Ltd. (Nanning, China). The HiScript^®^ III RT SuperMix for qPCR (+gDNA wiper) kit was used for the measurements. The total reaction system was 10 μL and consisted of a 0.5 μL cDNA template, 0.195 μL each of the forward and reverse primers, 4.885 μL of 2 × ChamQ SYBR qPCR Master Mix, 0.195 μL of 50 x ROX Reference Dye 1, and 4.03 μL pure water. The qRT-PCR program (CFX96) used was as follows: 95 °C for 3 min; 95 °C for 10 s and 60 °C for 30 s for a total of 39 cycles. This was followed by a melting curve analysis, starting at 65 °C in steps of 5 s until 95 °C was used with a final step at that temperature for 50 s. Each sample had three replicates, and the relative expression was calculated using the 2^−ΔΔCt^ method.

## 5. Conclusions

We performed metabolomics and transcriptomics analysis of the roots, stems, leaves, and flowers of *E. sonchifolia* in order to explore the transcriptional regulation mechanism of differential accumulation of flavonoids. The differential metabolites of flavonoids in the different tissues were identified, and key enzyme genes closely related to differential accumulation of flavonoids were evaluated. Our results will provide a reference for the functional expression analysis of *E. sonchifolia* and lay a foundation for improving the production of flavonoids in these medicinal plants. This study provides a theoretical basis for the cultivation of *E. sonchifolia* with increased efficacy and higher medicinal value.

## Figures and Tables

**Table 1:** The unigene annotation results obtained from each database.

Database	Number of Genes	Percentage (%)
KEGG	65,770	54.86
Nr	84,534	70.51
Swiss Prot	63,443	52.92
TrEMBL	84,466	70.46
KOG	54,675	45.61
GO	75,328	62.83
PFAM	64,036	53.41
Annotated in at least one database	89,576	74.72
Total unigenes	119,886	100

## Data Availability

The sequence data supporting the results of this study have been stored in the NCBI database. The main accession number is PRJNA1189669, and the website is https://www.ncbi.nlm.nih.gov/bioproject/?term=PRJNA1189669 (accessed on 26 May 2026).

## Supplementary Materials

The following supporting information can be downloaded at https://www.mdpi.com/article/10.3390/ijms27156803/s1.
