# Supplementary material for: Metabolomics and Transcriptomics Studies of the Differential Accumulation of Flavonoids in Different Organs in Emilia sonchifolia
Source: Int J Mol Sci. 2026 Jul 29;27(15):6803. doi: 10.3390/ijms27156803 (PMC13466634; doi:10.3390/ijms27156803)
Supplement: Supplementary file 1 [file ijms-27-06803-s001.zip › Supplement chart information.pdf]

Supplementary Figure S1

Statistics of flavonoids metabolites

Supplementary Figure S2

Difference of Flavonoids Metabolites in Different Parts of Emilia sonchifolia

Supplementary Figure S3

The first 20 material histograms of differential metabolites between different groups. (**A**): CBh3 vs CBr3; (**B**): CBh3 vs CBs3; (**C**): CBh3 vs CBl3; (**D**): CBr3 vs CBs3; (**E**): CBr3 vs CBl3; (**F**): CBs3 vs CBl3

Supplementary Table S1

Primer sequences of candidate genes and target genes

Supplementary Table S2

Flavonoids metabolites and their contents of *E. sonchifolia*

Supplementary Table S3

Statistics of sample sequencing data

Supplementary Table S4

Differentially expressed genes in flavonoid metabolic pathways of *E.sonchifolia*

Supplementary Table S5

Information on internal standard compounds used
